# Cardiac Telerehabilitation Using a Smartwatch and a Gamified Smartphone App: Single-Arm Pre-Post Feasibility Study

**DOI:** 10.2196/85808

**Published:** 2026-06-11

**Authors:** Yusaku Arima, Toshiki Kaihara, Megumi Nakamura, Keita Sone, Toshiya Yoshida, Yui Utsugi, Shiori Takizawa, Shunichi Doi, Kei Honda, Yoshikuni Kobayashi, Akira Kasagawa, Yasuhito Kawagoe, Takahiko Kai, Masashi Koga, Takumi Higuma, Yasuhiro Tanabe, Yoshihiro Akashi

**Affiliations:** 1 Department of Cardiology, St. Marianna University School of Medicine, Sugao 2-16-1, Miyamae-ku, Kawasaki, 2168511, Japan, 81 44-977-8111, 81 44-977-9486

**Keywords:** cardiac telerehabilitation, smartphone app, wearable technology, gamification, digital health, acute coronary syndrome

## Abstract

**Background:**

Home-based cardiac rehabilitation (CR) using digital health technologies (ie, cardiac telerehabilitation [CTR]) has emerged as a practical alternative to conventional center-based CR, particularly during and after the COVID-19 pandemic. However, maintaining sustained participation in CR remains challenging. Gamification holds the potential to enhance motivation and adherence in CR, but its role in CTR for patients with acute coronary syndrome (ACS) remains under-studied.

**Objective:**

This feasibility study evaluated the feasibility, acceptability, and safety of a combination of a gamification-enabled smartphone app and a smartwatch supporting CTR after ACS. We focused specifically on participation motivation, adherence to prescribed exercise intensity, and short-term physiological outcomes.

**Methods:**

This single-arm, pre-post intervention study was conducted at 2 Japanese institutions. Sixteen patients diagnosed with ST-elevated myocardial infarction or non–ST-elevated myocardial infarction and undergoing percutaneous coronary intervention were enrolled after discharge. Each patient received a smartphone and smartwatch connected to the Shin-po Kei app, through which participants earned points when exercising within their target anaerobic threshold heart rate (HR) range (±10 bpm). The 1-month intervention prescribed walking for 30 minutes or longer 3 to 5 days or more per week, at an intensity corresponding to a Borg scale score of 11 to 13. The primary end point was the number of app log-in days divided by the number of intervention days (app use rate). Secondary end points included the target HR exercise adherence rate (defined as the proportion of patients who exercised for 30 minutes within the target HR range on 3 or more days per week), changes in peak oxygen consumption, and changes in perceived exertion using a visual analog scale.

**Results:**

The mean patient age was 62 (SD 13) years, and 94% (15/16) were male. The mean intervention duration was 29 (SD 6) days, and the overall app use rate was 59.9%. The target HR exercise adherence rate was 50%. Peak oxygen consumption increased from 18.4 (SD 4.1) to 21.1 (SD 5.4) mL/kg/min, and the mean visual analog scale score for exercise-related hassle reduced from 57 (SD 33) to 32 (SD 26). No adverse events related to app use were identified, and 1 hospitalization unrelated to the intervention occurred during the study period.

**Conclusions:**

This feasibility study suggests that short-term engagement with a smartwatch-linked gamified smartphone app is achievable in CTR after ACS. However, adherence to predefined gamified exercise standards was lower than app use rates, suggesting that refinement of reward rules and target HR thresholds may be necessary.

## Introduction

Home-based cardiac rehabilitation (HBCR) is emerging as a feasible and effective alternative to traditional center-based cardiac rehabilitation (CBCR) for patients with cardiovascular disease [1,2]. HBCR achieves improvements in exercise capacity, quality of life, and management of cardiovascular risk factors comparable to those achieved with CBCR while offering enhanced convenience, reduced burden of hospital travel, and improved access, particularly for older adults and residents of rural or medically underserved areas [1]. Furthermore, HBCR is associated with high patient satisfaction and has the potential to reduce health care costs [1,3]. However, the participation rate in CBCR in Japan remains low, with reports indicating that only approximately 3% to 9% of eligible patients participate, suggesting that barriers, including issues related to accessibility, persist [4,5]. Contributing factors include a shortage of facilities offering cardiac rehabilitation (CR), low awareness among patients and health care providers, and geographical barriers such as remote islands and mountainous regions [6]. These barriers hinder sustained participation in hospital-based programs and limit the reach of conventional CR. To overcome these challenges, HBCR with telemedicine-based approaches—collectively termed cardiac telerehabilitation (CTR)—was developed. CTR uses digital health technologies such as smartphones, wearable devices, videoconferencing, and internet-connected ergometers to provide remote exercise coaching, telemonitoring, patient education, and lifestyle support [7]. Evidence suggests that CTR can achieve outcomes noninferior to traditional CR in terms of compliance, hospitalization rates, and long-term maintenance of physical activity.

The use of smartphone apps (mobile health [mHealth]) in cardiology is rapidly expanding, with promising results. For example, blood pressure management apps have demonstrated significantly superior blood pressure control compared with standard care [8]. Smartwatches recording chest electrocardiograms show high similarity to conventional 12-lead electrocardiograms [9]. A recent randomized controlled trial (RCT) found that a smartphone app linked to a Bluetooth-connected monitoring device improved dyspnea symptoms in patients with heart failure (HF) [10]. However, few studies have evaluated smartphone apps incorporating “gamification” [11]—integration of game elements into CR—for managing coronary artery disease.

A critical challenge is whether users will consistently use the app at the intended frequency. Continuous use is essential for achieving health benefits, but ensuring adherence is difficult. Gamification elements such as points and rewards may enhance motivation and contribute to reducing cardiovascular risk [12]. Smartphone apps incorporating reward systems directly linked to exercise around the anaerobic threshold heart rate (ATHR) have been virtually nonexistent. Although gamification approaches targeting step goals have been reported [13], few studies have incorporated physiological target values obtained from cardiopulmonary exercise testing (CPET) into CR using gamification. Previous research [14] reported a virtual reality–based CR program for patients with HF. This program monitored heart rate (HR) in real time, automatically adjusted exercise intensity, and maintained patients within target HR zones. Another gamification study [15] used avatars representing patients’ hearts and tokens that could be earned by addressing risk factors. However, in these studies, the “reward” element—a key component of gamification—was not explicitly linked to achieving specific HR thresholds.

This study’s intervention (a smartphone app incorporating gamification elements) is expected to enhance patient behavior change and demonstrate the app’s clinical significance by establishing a motivational mechanism. This is achieved by linking the “reward” component of gamification to individualized physiological targets derived from CPET—specifically, the ATHR—thereby integrating it with established principles of exercise prescription. This feasibility study explores the applicability of a gamified smartphone app combining ATHR-based point-earning functionality with HR monitoring via a smartwatch for CR. It specifically focuses on evaluating its acceptability and procedural feasibility as a foundation for future large-scale intervention development.

## Methods

### Study Design

This prospective single-arm feasibility study aimed to evaluate the usefulness of a gamified smartphone app in CTR. Sixteen patients with ST-elevated myocardial infarction (STEMI) or non-STEMI were enrolled and underwent CTR using a smartphone app and smartwatch immediately after discharge. The app incorporated gamification features, allowing users to earn points for exercise performed within an appropriate HR range and providing feedback based on the points earned.

### Recruitment

Patients were recruited from 2 facilities in Kawasaki City, Japan (St. Marianna University Hospital and Kawasaki Municipal Tama Hospital). Eligible patients were those diagnosed with STEMI or non-STEMI based on the criteria of the Japanese Circulation Society 2018 Guideline on Diagnosis and Treatment of Acute Coronary Syndrome [16], who underwent coronary intervention, routinely used a smartphone, and were able to walk outdoors without assistance. All patients received a written explanation of the study protocol and provided written informed consent prior to enrollment. Exclusion criteria included inability to provide consent, severe disease (eg, cancer or collagen vascular disease), or mobility impairment requiring assistance.

### Intervention

**Figure 1.**
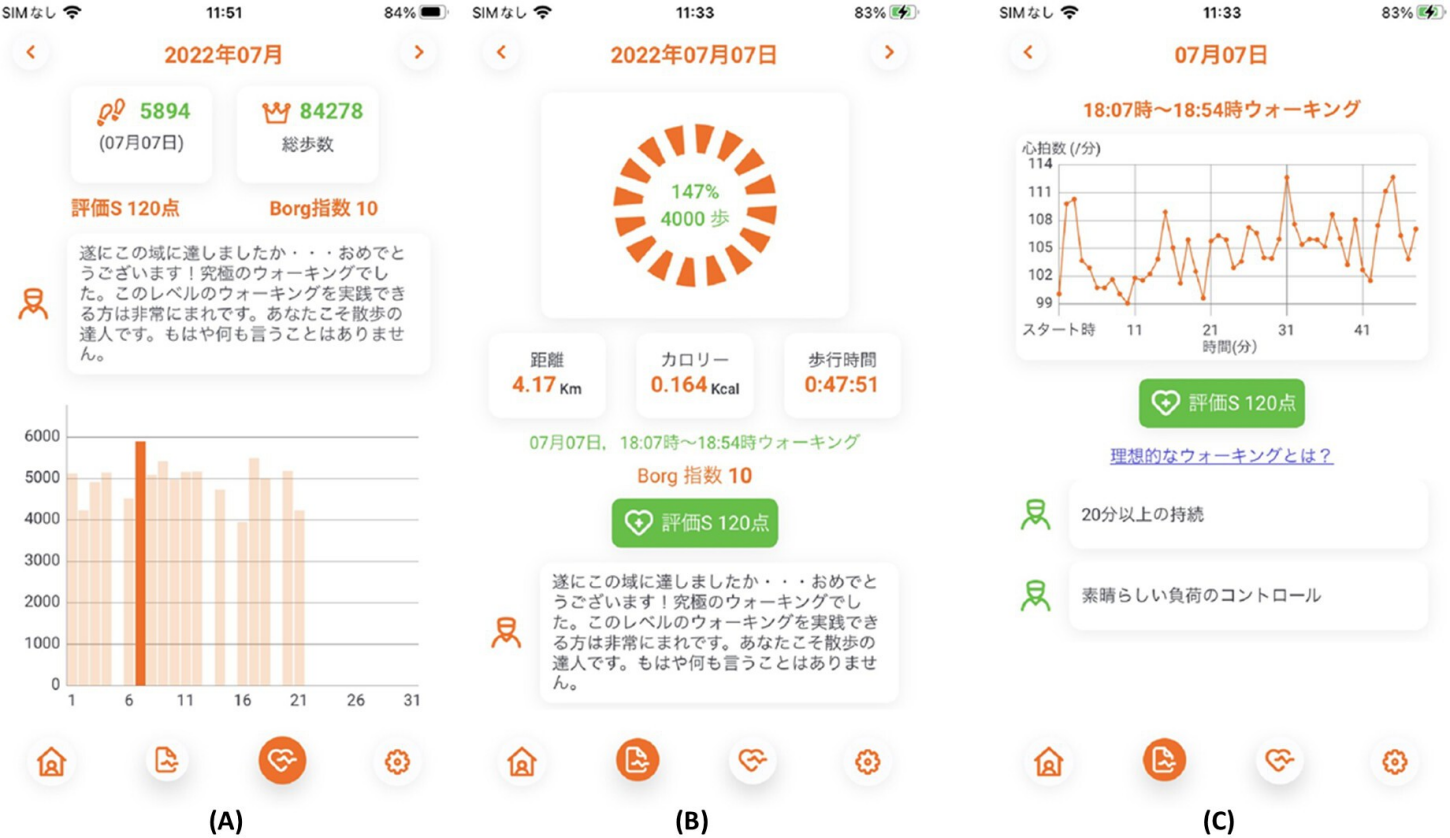
Shin-po Kei app screenshots. (A) The first screen shows the number of steps (daily and cumulative), the Borg scale score, and a step count histogram; (B) the second screen displays exercise distance, calories burned, and exercise time; and (C) the third screen shows a line graph with heart rate (bpm) on the y-axis and exercise time (minutes) on the x-axis. Each screen also shows the points earned for the day, along with an automatically generated comment based on the points.

Patients in the intervention group participated in the CTR program after discharge. This program included warm-up and cool-down stretches, aerobic exercise, and resistance training, which were explained in advance by physicians and physical therapists prior to discharge. The aerobic exercise component was supported by digital devices. Patients were loaned an iPhone 7 (Apple Inc) and an Apple Watch Series 3 (Apple Inc). The Shin-po Kei app was installed on the smartphone and paired with the smartwatch via Bluetooth. Patients were instructed to perform stretching and resistance training, plus aerobic exercise consisting of walking for at least 30 minutes (Borg scale score 11‐13) on 3 to 5 days or more per week, based on the Japanese Circulation Society–Japanese Association of Cardiac Rehabilitation 2021 Guideline on Rehabilitation in Patients with Cardiovascular Disease [17]. For the Borg scale, patients manually entered their scores into the app after each exercise session. Since the first day of the study period coincided with the date of discharge and the last day coincided with the first outpatient visit following discharge (1 month later), 2 days were subtracted from the period calculated based on the dates to determine the intervention period. The app recorded HR and exercise duration, awarding points for exercise within the target HR range (ATHR ±10 bpm). Four points were awarded for each minute spent within the target HR range, allowing a maximum of 120 points for a 30-minute exercise session. Points were deducted at a rate of 2 points per minute for exceeding the target range. These precautions were implemented because patients were immediately after discharge and safety was the top priority. Patients could directly view their daily achievements and progress on the app through automatically generated feedback messages and visual data (eg, line graphs; Figure 1 shows screenshots and Multimedia Appendix 1 provides a translation of the screenshots into English). Regarding step count, although it was displayed in the window, no further feedback was provided. Feedback was provided after completion of the walk (ie, after the patient sent the data via the smartphone app). The underlying logic for the automatic feedback messages was based on points earned (ie, HR during exercise and exercise duration).

### Outcome Measures and Data Collection

The primary end point was the proportion of days during the period between discharge and the first outpatient visit on which an intervention occurred (ie, the patient logged into the app). “App use” was defined as “logging into the app” and was assessed exclusively using app-recorded data automatically collected and verified by the app and smartwatch systems, without reliance on self-reported measures.

Secondary end points included the proportion of patients who exercised for 30 minutes within the target HR range on 3 or more days per week using the smartphone app during the period from discharge to the first outpatient visit (target HR exercise adherence rate), and changes in peak oxygen consumption (VO_2_), low-density lipoprotein cholesterol, hemoglobin A_1c_, and depression or anxiety symptoms (Hospital Anxiety and Depression Scale). Peak VO_2_ and anaerobic threshold were measured using symptom-limited CPET and the V-slope method. CPET was performed using a cycle ergometer (SE-8; Mitsubishi Electric Engineering Corp) and a respiratory gas analyzer (Minato Medical Science Co Ltd). The cycle ergometer exercise protocol included a 0-W warm-up and a 10-W/min incremental load. Preintervention CPET was conducted at the start of the study, and postintervention CPET was conducted at the end of the study. Additional secondary end points included changes in exercise-related hassle (visual analog scale [VAS] with 0-100 scoring, where 0 indicated no hassle and 100 indicated extreme hassle), which was designed to evaluate psychological burden and perceived inconvenience associated with exercise rather than physiological symptoms such as pain or shortness of breath; correlations between type D personality (Type D Scale-14) and the primary end points, and the incidence of new complications. This complication assessment was performed by physicians (outpatient physicians) independent of the study investigators. A questionnaire regarding app use was also administered to assess the app’s contribution to exercise, reasons for exercise discontinuation, inconveniences encountered during app use, and potential gamification elements that could enhance motivation for aerobic exercise if incorporated in the future. These usability and feasibility assessments were conducted using app use rates and study-specific questionnaires developed for this pilot study.

### Statistical Analysis

Statistical analyses were performed using R software (version 4.3.2; R Foundation for Statistical Computing). Data are presented as mean (SD), median (IQR), or percentage. The chi-square test was used for proportions, paired *t* tests for pre-post comparisons, and Pearson or Spearman correlation coefficients for parametric and nonparametric data, respectively. An intention-to-treat analysis was performed. A 2-tailed *P*<.05 was considered statistically significant. On the basis of prior research [18,19], the pilot study sample size was calculated as 10% of the projected sample size for the larger RCT (150 patients), requiring at least 15 patients. The sample size was determined based on the feasibility of conducting the pilot study, rather than on a power analysis designed to detect a specific effect size.

### Ethical Considerations

The protocol, informed consent form, and other research-related documents for this study were approved by the Ethics Committee of St. Marianna University School of Medicine (approval 5512; UMIN 000046777). This study was conducted in accordance with the Declaration of Helsinki and Good Clinical Practice guidelines. Written informed consent was obtained from all patients prior to enrollment. The initial informed consent approved the study procedures without requiring additional consent for secondary analyses. To ensure patient privacy and confidentiality, all study data were anonymized and deidentified prior to analysis. No financial compensation was provided for participation in this study. The manuscript and supplementary materials contain no images or information that could identify individual patients.

## Results

From April to August 2022, a total of 36 consecutive patients met the inclusion criteria, of whom 16 patients enrolled. Overall, 8 patients were admitted to St Marianna University Hospital, and 8 patients were admitted to Kawasaki Municipal Tama Hospital. Table 1 shows the baseline characteristics of the patients. The mean age was 62 (SD 13) years, and 94% (n=15) were male. STEMI accounted for 81% (n=13) of cases. The mean intervention period was 29 (SD 6) days, and the mean length of hospital stay was 12 (SD 1) days. Among the 20 patients who declined participation, the most common reason for refusing consent was “not being able to use a smartphone (not having a smartphone).” The results are summarized in Table S1 in Multimedia Appendix 1.

**Figure 2.**
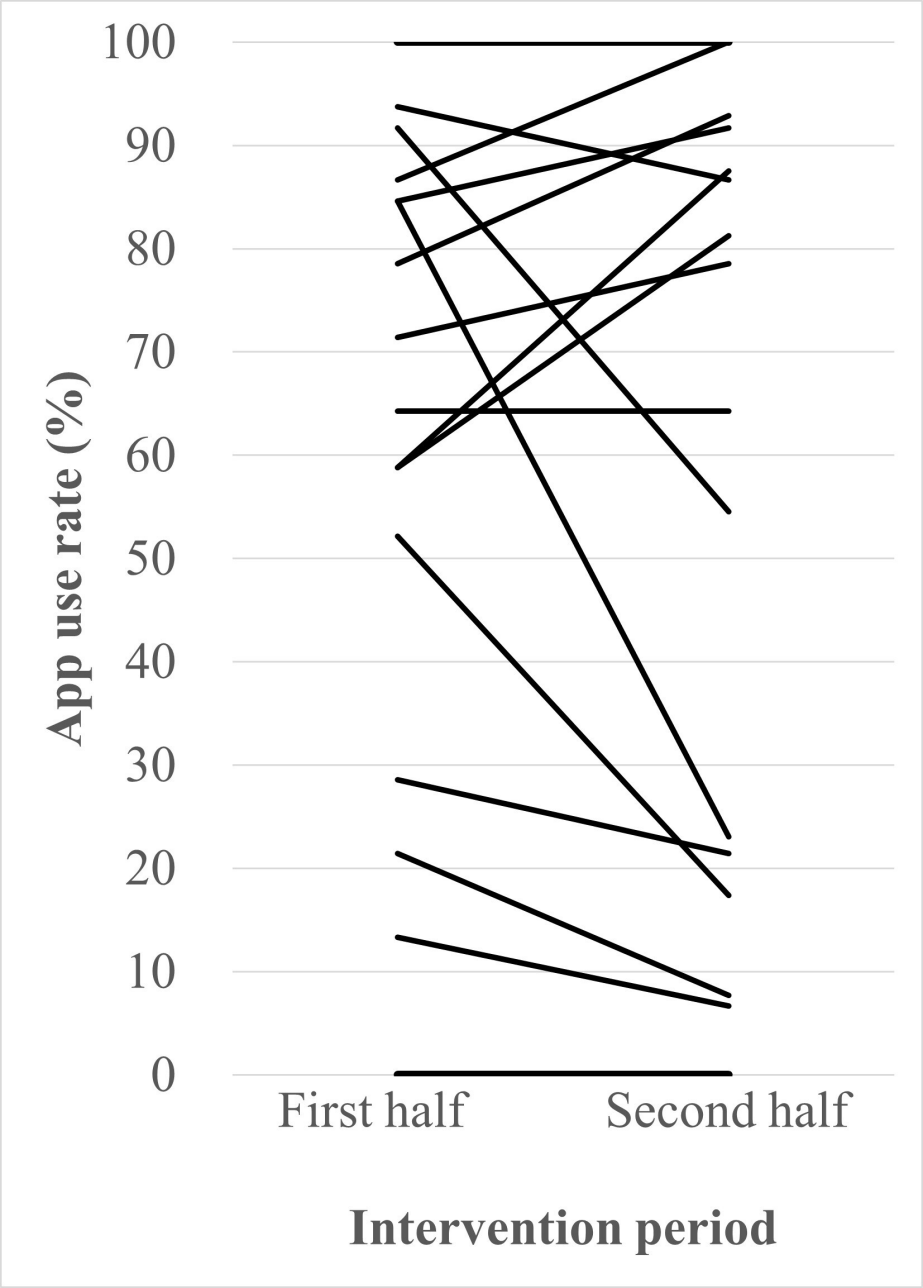
App use rates over time for each patient.

The overall app use rate (number of log-in days or number of intervention days) was 59.9% (282/471 days). The median app use rate was 71.5% (IQR 32.5%-86.5%; detailed data is shown in Table S2 in Multimedia Appendix 1). The rate was 63.1% (149/236 days) in the first half of the intervention period and 56.6% (133/235 days) in the second half (Figure 2) .

**Table 1.** Baseline characteristics of the study participants (N=16).

Characteristic	Participants
Age (years), mean (SD)	62 (13)
Male sex, n (%)	15 (94)
BMI (kg/m^2^), mean (SD)	25.4 (4.0)
Smoking status, n (%)	
Current	5 (31)
Former	6 (38)
Never	4 (25)
Not documented^a^	1 (6)
Diagnosis, n (%)	
ST-elevated myocardial infarction	13 (81)
Non–ST-elevated myocardial infarction	3 (19)
Left ventricular ejection fraction ≥50%, n (%)	12 (75)
Laboratory data, median (IQR)	
Low-density lipoprotein cholesterol (mg/dL)	116 (101-127)
Estimated glomerular filtration rate (mL/min/1.73 m^2^)	58 (52-69)
Medication data, n (%)	
β-blockers	15 (94)
Angiotensin-converting enzyme inhibitors, angiotensin II receptor blockers, or angiotensin receptor neprilysin inhibitors	12 (75)
Statins	16 (100)
Cardiopulmonary exercise testing data, mean (SD)	
Peak oxygen consumption (mL/kg/min)	18.4 (4.1)
Anaerobic threshold heart rate (bpm)	93 (11)
Intervention data, mean (SD)	
Length of hospital stay (days)	12 (1)
Intervention duration (days)	29 (6)

a Not recorded in the patient’s medical record.

For secondary end points, the target HR exercise adherence rate (defined according to the Japanese Circulation Society–Japanese Association of Cardiac Rehabilitation guidelines [17] and excluding the aforementioned penalties) was 50% (8/16 patients), and the average frequency of exercise sessions lasting 30 minutes was 2.9 (SD 2.1) days per week. The rate of exercise sessions that scored 120 points on the app was 32.9% (155/471 days). Furthermore, the rate of exercise sessions lasting ≥30 minutes within the target HR range, calculated without taking penalties (ie, points deducted for exceeding the target HR range) into account, was 42.0% (198/471 days). Peak VO_2_ showed a statistically significant increase from 18.4 (SD 4.1) to 21.1 (SD 5.4) mL/kg/min (*P*=.004), and the mean VAS score for exercise-related hassle showed a statistically significant reduction from 57 (SD 33) to 32 (SD 26; *P*=.007). No significant differences were observed in preintervention and postintervention laboratory data (low-density lipoprotein cholesterol and hemoglobin A_1c_) or Hospital Anxiety and Depression Scale scores. No significant correlation was found between Type D Scale-14 scores and app use rates (*r*=0.007; *P*=.98; Figure S2 in Multimedia Appendix 1). One patient did not participate in the intervention, but the data were included in the analysis in accordance with the intention-to-treat principle. One patient was hospitalized during the study period because of severe anemia caused by prolonged prothrombin time-international normalized ratio; however, this event was recognized by the outpatient physician to be the result of inadequate anticoagulant dose adjustment, and no adverse events related to app use were reported (Table S3 in Multimedia Appendix 1).

Survey responses revealed the following: in terms of assessing an app’s contribution to exercise, “display of results” (eg, step count, HR, and points) was the most common response (4 patients). In terms of assessing reasons for exercise discontinuation, reasons included “hot weather” (3/16, 19%), “effort required for exercise” (n=3, 19%), and “hospitalization” (n=1, 6%). In terms of assessing inconveniences encountered during app use, 14 patients responded, “none in particular”; 1 patient each cited “discrepancy between points and self-assessment” and “smartwatch battery drain.” Regarding potential gamification elements that could enhance motivation for aerobic exercise if incorporated in the future, 14 patients responded. When presented with a list of potential gamification elements, the most frequently selected options were “level system (levels increase with points),” selected by 9 patients, and “calendar display (allows quick daily progress checks in calendar format),” also selected by 9 patients. The next most common responses were “monetary rewards,” selected by 6 patients, “nonmonetary rewards,” selected by 4 patients, and “character development” (allowing users to improve a character within the app; eg, earn points through exercise therapy to make the character stronger), selected by 4 patients. The detailed responses are shown in Table S4 in Multimedia Appendix 1.

## Discussion

### Principal Findings

This feasibility study found that the smartphone app use rate (number of log-in days or number of intervention days) was 59.9% over 1 month, suggesting that the gamification app showed moderate short-term uptake during the 4-week intervention; however, causal relationships cannot be inferred from this study. Furthermore, an increase in peak VO_2_ was observed during the intervention period, and no significant safety concerns were identified with the use of the gamified app in patients after acute coronary syndrome.

### Comparison to Prior Work

Previous studies on digital health, gamification, and CTR [13,15,20] have reported engagement or adherence rates of approximately 30% to 70%. Furthermore, real-world data indicate that the continued use rate of general health apps often drops to approximately 10% after 30 days [21]. Although strict numerical criteria for feasibility were not predefined in this study, the observed 1-month app use rate of 59.9% falls within the range reported in previous studies. However, a tendency toward declining use was observed in the latter part of the intervention period. This aligns with prior findings indicating that sustained engagement with mHealth apps becomes challenging over time. Previous reports attribute this decline to diminishing novelty, competing daily demands, reduced intrinsic motivation, and technical issues such as device synchronization and battery depletion [21]. Our findings may reflect these common barriers to digital health adoption.

To prevent declining app use rates, based on survey findings regarding patient motivation, implementing a calendar view that allows results to be confirmed at a glance and introducing a “level” system—a type of nonmonetary reward obtained by accumulating a certain number of points—may be worth considering. Furthermore, on the basis of survey findings indicating that “visualizing results is important” for supporting the establishment of regular exercise habits, “automatic” result displays may also represent an effective strategy (Multimedia Appendix 1). Studies investigating CR with app or home-based system follow-up have demonstrated improvements in peak VO_2_ and health-related quality of life, with some showing significant gains at 1 year and others confirming effects at 12 weeks [22,23]. This study demonstrated similar trends, with improvements in peak VO_2_. The observed increase exceeded the minimum clinically meaningful difference of 1 mL/kg/min for reducing mortality risk [24]. Additionally, exercise-related hassle (VAS score) improved.

### Gamification

Gamification has been shown to enhance motivation and physical activity levels [13,25], and a recent meta-analysis reported its beneficial effects on glycemic control in type 2 diabetes through improved physical activity [12]. The Shin-po Kei app uses a gamification technique involving earning and losing points based on HR, which may have contributed to the favorable outcomes. This mechanism can be interpreted within a behavioral change framework: points act as nonmonetary informational rewards that provide immediate feedback and support self-monitoring, feedback, and goal-setting strategies [26]. Meta-analyses show that gamification elements such as points and levels increase physical activity and improve clinical outcomes by enhancing motivation even without financial incentives [27]. These elements deliver continuous feedback that reinforces behavior by fulfilling the core psychological needs of competence and autonomy [28].

CR is fundamentally an intervention designed to promote behavioral change. Although evidence linking reward systems to objective measures of aerobic capacity remains limited, we believe that the introduction of reward systems—a component of gamification also used to promote behavioral change—has high affinity with CR. However, exercise intensity (ie, exercise within target HR zones) was not optimal. This is likely due to the app’s safety rules penalizing exercise that exceeded the upper HR limit, which may have been set too low for patients with mild myocardial ischemia (refer to the response regarding inconveniences encountered during app use). This may have contributed to a decrease in patients’ exercise adherence rates. Although we stated that “peak VO_2_ significantly increased from 18.4 (SD 4.1) mL/kg/min,” the actual range was quite broad, from 10.6 to 25.6 mL/kg/min. Gamification rules should be individually set based on clinical stability and physical ability. Rather than eliminating the upper HR limit, future adjustments to the target HR zone should be considered. Expanding the acceptable HR range or introducing dynamically adjusted thresholds based on repeated clinical evaluations may allow greater flexibility while maintaining load management, a fundamental principle of CR. Patient safety remains the paramount priority in any modification to exercise prescriptions based on gamification. Alternatively, applying the system to patient groups for whom exercise exceeding the anaerobic HR threshold poses a low safety risk—such as those with stable angina—could be explored. Such adjustments could maintain safety while enabling more flexible exercise behavior, potentially improving exercise adherence.

### Safety

During the 4-week study period, 1 patient was hospitalized, resulting in a temporary interruption of exercise. The cause of hospitalization, as detailed in the Results section, was unrelated to the intervention. No adverse events directly attributable to app use or the exercise prescription were identified, supporting the safety of the mobile CR app. Other gamification studies also report no clear harm [13,15]; therefore, the use of smartphone apps in CR appears to be generally safe. Regarding the relationship between data privacy and ethical issues in mHealth, as explored in this study, concerns such as “secondary data use for commercial purposes” and “psychological constraints arising from monitoring” exist, indicating numerous challenges that require resolution in the future.

### Limitations

This feasibility study has several limitations. First, the sample size was small, and the efficacy of gamified smartphone apps should be evaluated in larger RCTs. In the absence of a control group, the observed improvements in peak VO_2_ and VAS scores may be attributable, at least in part, to natural physiological recovery during the early postmyocardial infarction phase rather than to the intervention itself.

Second, as mentioned, participation rates were low, potentially introducing selection bias. Furthermore, this study included a predominantly male population (94%), which may limit the generalizability of the findings to female patients. As noted in a recent American Heart Association scientific statement [29], women are known to have lower participation rates in CR programs due to multifactorial barriers. Therefore, caution should be exercised when extrapolating these feasibility findings to female patients. In addition, as “routine smartphone use” was set as the inclusion criterion, patients affected by the digital divide—such as older adults, those without smartphones (the primary reason for exclusion), or those without stable internet access—may have been excluded, potentially limiting the study’s generalizability. The same applies to patients with HF; there were almost no patients with concomitant HF in this study, and even when considering the existing study on mHealth [20], the conclusions remain controversial. However, the prior research [30] has highlighted the importance of “digital health readiness” in addressing this issue. This study did not collect data on patients’ educational attainment or digital literacy. These factors may influence the feasibility of CTR and the level of engagement with digital health interventions. Future research should consider introducing tools such as digital health readiness questionnaires to further enhance patient engagement.

Third, although the app can be installed on patients’ personal iPhones, it was developed exclusively for iOS because of budgetary constraints, which may have affected the generalizability of the findings. To avoid exclusion of patients based on device type, all patients in this study were uniformly provided with an iPhone and an Apple Watch. However, this device-loaning model may be economically challenging to implement in clinical practice. A bring-your-own-device approach, in which patients install the app on their own smartphones, may offer greater cost-effectiveness and user satisfaction. In the subsequent study (the GamiHeart II study), a bring-your-own-device design has been adopted to enhance feasibility and real-world implementability. Furthermore, the devices used in this study were not the latest models at the time of intervention. Therefore, technical inconveniences such as battery drain may partially reflect hardware-related limitations rather than inherent flaws of the software intervention itself. Furthermore, missing data related to hardware functionality may have contributed to the underestimation of exercise adherence based on target HR (Table S2 in Multimedia Appendix 1). Future evaluations using next-generation devices may yield different usability results.

Fourth, although gamification is an integral component of the app used in this study, it is not an isolated feature but is closely intertwined with other functionalities. For example, feedback messages in this study are commonly seen in smartphone apps and vary greatly in nature, ranging from those similar to what physicians provide to patients in routine clinical practice to those designed to enhance motivation for earning monetary or nonmonetary rewards within the app. Separating the gamification elements of this app from other aspects of health care can sometimes be difficult. Although creating an app linked to more “game-like” elements, such as puzzle games, might allow measurement of gamification capabilities, the results observed in this study likely reflect the combined effect of the entire app, including its gamification features.

Fifth, another important limitation of this study is the relatively short intervention period of approximately 4 weeks. Standard CR programs are typically conducted over 12 weeks or longer; therefore, this study duration is insufficient to adequately evaluate sustained participation or long-term adherence to the intervention. For 1 patient, the hospitalization period was included in the intervention period, so this patient’s adherence period may have been underestimated. Notably, a decline in app use was observed during the latter half of the study period, suggesting that patient engagement may decrease over time. Future, longer-term, large-scale RCTs are warranted to evaluate adherence, cost-effectiveness, and long-term clinical outcomes of gamified CTR.

Finally, usability and acceptability were not evaluated using established, validated measures such as the System Usability Scale or the mHealth App Usability Questionnaire. This limits direct comparisons with digital health interventions in other studies.

### Conclusions

This feasibility study suggests that short-term engagement with mHealth using a gamified smartphone app for CTR after acute coronary syndrome is achievable. However, adherence to predefined gamified exercise criteria was lower than app use rates, indicating a potential need for refinement of reward rules and target HR thresholds. These findings warrant further evaluation in larger-scale studies.

## Supplementary material

10.2196/85808 Multimedia Appendix 1 Supplementary tables detailing consent refusal reasons, patient app use rates, adverse events, questionnaire responses on exercise motivation, and a translation and summary of the screenshots in Figure 1.

## Abbreviations

ATHR: anaerobic threshold heart rate
CBCR: center-based cardiac rehabilitation
CPET: cardiopulmonary exercise testing
CR: cardiac rehabilitation
CTR: cardiac telerehabilitation
HBCR: home-based cardiac rehabilitation
HF: heart failure
HR: heart rate
mHealth: mobile health
RCT: randomized controlled trial
STEMI: ST-elevated myocardial infarction
VAS: visual analog scale
VO_2_: oxygen consumption
