# Supplementary material for: Cardiac Telerehabilitation Using a Smartwatch and a Gamified Smartphone App: Single-Arm Pre-Post Feasibility Study
Source: JMIR Cardio. 2026 Jun 11;10:e85808. doi: 10.2196/85808 (PMC13256502; doi:10.2196/85808)
Supplement: Multimedia Appendix 1 [file cardio-v10-e85808-s001.docx]

**Supplementary Table 1. Reasons for refusing consent**

| **Reasons for Refusing Consent** | **No.** |
| --- | --- |
| Not being able to use a smartphone (not owning a smartphone) | 13 |
| Inability to communicate due to the severity of the clinical condition | 2 |
| Inability to communicate due to intellectual disability | 1 |
| Clinically rejected by a physician |  |
| Difficulty in setting the target heart rate due to atrial fibrillation | 1 |
| Difficulty in setting the target heart rate due to pacemaker rhythm | 1 |
| Mobility impairment | 1 |
| Hassle | 1 |
| **Total** | **20** |

**Supplementary Table 2. Detailed app usage rate for each patient**

| **Study ID** | **No. of intervention days** | **No. of login days** | **App usage rate** | **No. of days with significant gaps in HR logs despite being logged in** |
| --- | --- | --- | --- | --- |
| 1 | 46 | 16 | 35 | 3 |
| 2 | 33 | 24 | 73 | 0 |
| 3 | 31 | 28 | 90 | 0 |
| 4 | 30 | 28 | 93 | 0 |
| 5 | 28 | 21 | 75 | 0 |
| 6 | 30 | 3 | 10 | 0 |
| 7 | 33 | 23 | 70 | 0 |
| 8 | 33 | 33 | 100 | 0 |
| 9 | 28 | 7 | 25 | 0 |
| 10 | 26 | 14 | 54 | 0 |
| 11 | 22 | 0 | 0 | 0 |
| 12 | 28 | 18 | 64 | 2 |
| 13 | 27 | 4 | 15 | 0 |
| 14 | 28 | 24 | 86 | 0 |
| 15 | 25 | 22 | 88 | 0 |
| 16 | 23 | 17 | 74 | 0 |

**Supplementary Table 3. All adverse events**

| **Study ID** | **Study start date** | **Study completion date** | **Adverse events** |
| --- | --- | --- | --- |
| 1 | 2022/4/14 | 2022/5/31 | N/A |
| 2 | 2022/4/20 | 2022/5/24 | N/A |
| 3 | 2022/5/21 | 2022/6/22 | N/A |
| 4 | 2022/5/14 | 2022/6/14 | N/A |
| 5 | 2022/5/24 | 2022/6/22 | N/A |
| 6 | 2022/5/29 | 2022/6/29 | N/A |
| 7 | 2022/6/18 | 2022/7/22 | N/A |
| 8 | 2022/6/21 | 2022/7/25 | N/A |
| 9 | 2022/6/23 | 2022/7/22 | Hospitalization  (2022/7/5-2022/7/15, due to severe anemia caused by prolonged PT-INR) |
| 10 | 2022/6/29 | 2022/7/26 | N/A |
| 11 | 2022/6/22 | 2022/7/15 | N/A |
| 12 | 2022/7/24 | 2022/8/22 | N/A |
| 13 | 2022/8/9 | 2022/9/6 | N/A |
| 14 | 2022/8/15 | 2022/9/13 | N/A |
| 15 | 2022/8/18 | 2022/9/13 | N/A |
| 16 | 2022/8/19 | 2022/9/12 | N/A |

**Supplementary Table 4-1. Detailed questionnaire responses for each patient (Q1-3)**

| **Study ID** | **Q1. "Which part of the app's system helped you stick with your exercise routine?"** | **Q2. "If there was a period during the month when you were unable to maintain your exercise routine, what was the reason?"** | **Q3. "Have you experienced any inconveniences while using the app or system?"** |
| --- | --- | --- | --- |
|  |  |  |  |
| 1 | The aspect that evaluates results regarding exercise (such as walking) | Work (weekdays) | None in particular |
| 2 | None in particular | None in particular | None in particular |
| 3 | The ability to verify results numerically | None in particular | The fact that the post-event evaluation doesn't match my own feelings |
| 4 | None in particular | None in particular | None in particular |
| 5 | None in particular | Housework and hot weather | None in particular |
| 6 | The fact that values such as averages and daily exercise levels are quantified | None in particular | None in particular |
| 7 | None in particular | The hassle of exercise | None in particular |
| 8 | None in particular | None in particular | None in particular |
| 9 | None in particular | Hospitalization | None in particular |
| 10 | None in particular | Loss of motivation to exercise | 1. Feeling pressured when feeling unwell or lacking energy 2. The smartwatch drains its battery quickly, so I can't wear it constantly, and sometimes I can't put it on when I want to |
| 11 | None in particular | Hot climate | None in particular |
| 12 | None in particular | None in particular | None in particular |
| 13 | The ability to check results such as heart rate | Hot climate | None in particular |
| 14 | None in particular | None in particular | None in particular |
| 15 | None in particular | The hassle of exercise | None in particular |
| 16 | None in particular | None in particular | None in particular |

**Supplementary Table 4-2. Detailed questionnaire responses for each patient (Q4)**

|  | | **Study ID** | | | | | | | | | | | | | | | |
| --- | --- | --- | --- | --- | --- | --- | --- | --- | --- | --- | --- | --- | --- | --- | --- | --- | --- |
|  |  | 1 | 2 | 3 | 4 | 5 | 6 | 7 | 8 | 9 | 10 | 11 | 12 | 13 | 14 | 15 | 16 |
| **Q4. "What kind of 'game elements' do you think would further increase your motivation for exercise therapy?"** | 1) Level system (levels increase with points) | ○ |  | ○ | ○ | ○ | ○ | ○ | ○ | ○ |  |  |  | ○ |  |  |  |
|  | 2) Character development (allowing users to grow a character within the app) |  |  |  | ○ |  |  | ○ | ○ |  |  |  |  |  |  | ○ |  |
|  | 3) “Role-playing” feature (apps with storylines, like role-playing games) |  |  |  |  |  |  |  |  |  |  |  |  | ○ |  |  |  |
|  | 4) “Event” feature (holding events such as “double points when participating in exercise therapy during a certain month”) |  |  |  | ○ |  |  | ○ |  | ○ |  |  |  |  |  |  |  |
|  | 5) Team Competition (conducting team competitions where patients compete based on their exercise therapy achievement levels [e.g., municipal competitions]) | ○ |  |  |  |  | ○ |  |  |  |  |  |  |  |  |  |  |
|  | 6) Ranking Chart (allowing patients undergoing exercise therapy to see their own “rank” and compare their situation with others) |  |  |  |  | ○ |  | ○ |  |  |  |  |  | ○ |  |  |  |
|  | 7) “Social” features (interacting with patients undergoing exercise therapy [via chat, message boards, etc.]) |  |  |  |  |  |  |  | ○ |  | ○ |  |  |  |  |  |  |
|  | 8) Non-monetary rewards |  |  |  | ○ |  |  | ○ |  |  |  |  |  | ○ |  | ○ |  |
|  | 9) Monetary rewards | ○ |  |  |  |  |  | ○ | ○ | ○ |  |  |  | ○ |  | ○ |  |
|  | 10) Calendar display (allowing quick daily progress checks in calendar format) |  |  | ○ | ○ | ○ | ○ |  | ○ |  | ○ | ○ | ○ | ○ |  |  |  |
|  | 11) Presence of “characters” (being praised or given advice on the results of exercise therapy by “characters” [e.g., mascot characters]) |  |  |  | ○ |  |  |  | ○ | ○ |  |  |  |  |  |  |  |

**Supplementary Figure 1. Japanese translation and summary of Figure 1**

1. Leftmost Image
The top row shows the daily step count (shown as July 7th in the figure) and the total step count during the study period. The second row shows the points earned for walking at that time (left side) and the Borg scale (right side). Below that, an automatically generated message appears designed to boost the patient's motivation based on the earned points (“You've finally reached this level... Congratulations! That was an excellent walk. It's truly wonderful that you can maintain this level of walking. There's nothing more to say”).

2. Center image
The top row shows the daily target step count and achievement rate. The middle row displays walking distance, calories burned, and walking time. The bottom section is identical to the content in “1. Leftmost image”.

3. Rightmost image
The top graph shows heart rate during walking (X-axis: walking time, Y-axis: heart rate). The bottom section displays an automatically generated message providing a simple analysis of this walk (“Walking for over 20 minutes” and “Excellent load control”).
